# Disruption of Dorsolateral Prefrontal Cortex Decreases Model-Based in Favor of Model-free Control in Humans

**DOI:** 10.1016/j.neuron.2013.08.009

**Published:** 2013-11-20

**Authors:** Peter Smittenaar, Thomas H.B. FitzGerald, Vincenzo Romei, Nicholas D. Wright, Raymond J. Dolan

**Affiliations:** 1 Wellcome Trust Centre for Neuroimaging, Institute of Neurology, UCL, 12 Queen Square, London WC1N 3BG, UK; 2 Centre for Brain Science, Department of Psychology, University of Essex, Wivenhoe Park, Colchester CO4 3SQ, UK

## Abstract

Human choice behavior often reflects a competition between inflexible computationally efficient control on the one hand and a slower more flexible system of control on the other. This distinction is well captured by model-free and model-based reinforcement learning algorithms. Here, studying human subjects, we show it is possible to shift the balance of control between these systems by disruption of right dorsolateral prefrontal cortex, such that participants manifest a dominance of the less optimal model-free control. In contrast, disruption of left dorsolateral prefrontal cortex impaired model-based performance only in those participants with low working memory capacity.

## Highlights

• Disrupting right dorsolateral prefrontal cortex impairs flexible model-based choice
• This drives behavior toward simpler, model-free control
• Results provide causal evidence for neural underpinnings of flexible choice

## Teaser

Choices are governed by computationally simple as well as complex systems that compete with each other. In this Report, Smittenaar et al. present causal evidence that the dorsolateral prefrontal cortex is required for such complex, goal-directed decision making.

## Introduction

Why is our behavior at times automatic and driven by habit and at other times deliberative and focused on a specific goal? Although most of us seamlessly switch between these modes of behavior, it has been suggested that a relative dominance of either habit-like or goal-directed modes of behavior underpin a range of disorders that span addictions (Everitt and Robbins, 2005) through to Parkinson’s disease (de Wit et al., 2011). This renders understanding the parsing of control between these two modes of decision making a pressing issue. Here we address whether it is possible to causally manipulate their relative dominance.

An elegant computational framework that captures the presence of (often competing) habit-like and goal-directed behaviors is provided by a formulation of model-free and model-based control (Daw et al., 2005, Dayan and Niv, 2008). A model-free system learns a single value for each action based on reward prediction errors and guides behavior based on these alone, thus trading a minimum of computational effort against the cost of a relative lack of flexibility in adjusting to current goals. Model-based control, by contrast, dynamically computes optimal actions by forward planning, a process that is computationally demanding but allows for flexible, outcome-specific behavioral repertoires (Daw et al., 2005, Dayan and Niv, 2008, Otto et al., 2013; but see Gershman et al., 2012).

In this study, our goal was to manipulate the relative balance between these two systems in human participants. We focused on the dorsolateral prefrontal cortex (dlPFC) as a substrate for model-based processes based on previous evidence for its role in the construction and use of associative models (Gläscher et al., 2010, Wunderlich et al., 2012a, Xue et al., 2012) and the coding of hypothetical outcomes (Abe and Lee, 2011). Work on nonhuman primates also implicates the dlPFC as a site for convergence of reward and contextual information (Lee and Seo, 2007), while lesions of rat prelimbic region (which some argue is equivalent to primate dlPFC [Fuster, 2008; but see Preuss, 1995, Uylings et al., 2003]) abolishes flexible decision making (Killcross and Coutureau, 2003).

Therefore, while the literature suggests a crucial role for this region in model-based control to date there is a lack of causal evidence to support this hypothesis. Here we used a transient lesion model, as engendered by theta burst transcranial magnetic stimulation (TBS), to provide evidence for a necessary role of dlPFC in model-based behavior.

## Results

### Transcranial Magnetic Stimulation and Task

We recruited 25 human participants (mean age [SD]: 24.2 [4.0] years; 15 females) to perform a task in which behavior can be explained by a mixture of model-free and model-based control (Daw et al., 2011). All participants were tested on three separate sessions (3 to 16 days apart) after MRI-guided TBS to the right dlPFC, left dlPFC, or vertex. TBS is known to inhibit cortical excitability for at least 20 min (Huang et al., 2005). We thus predicted that participants would show reduced model-based control after dlPFC compared to vertex TBS. Given existing evidence of functional asymmetries between left and right dlPFC, e.g., in reciprocal fairness (Knoch et al., 2006) and working memory (Mull and Seyal, 2001), we also hypothesized that the effects of TBS would differ between these sites.

**Figure 1:**
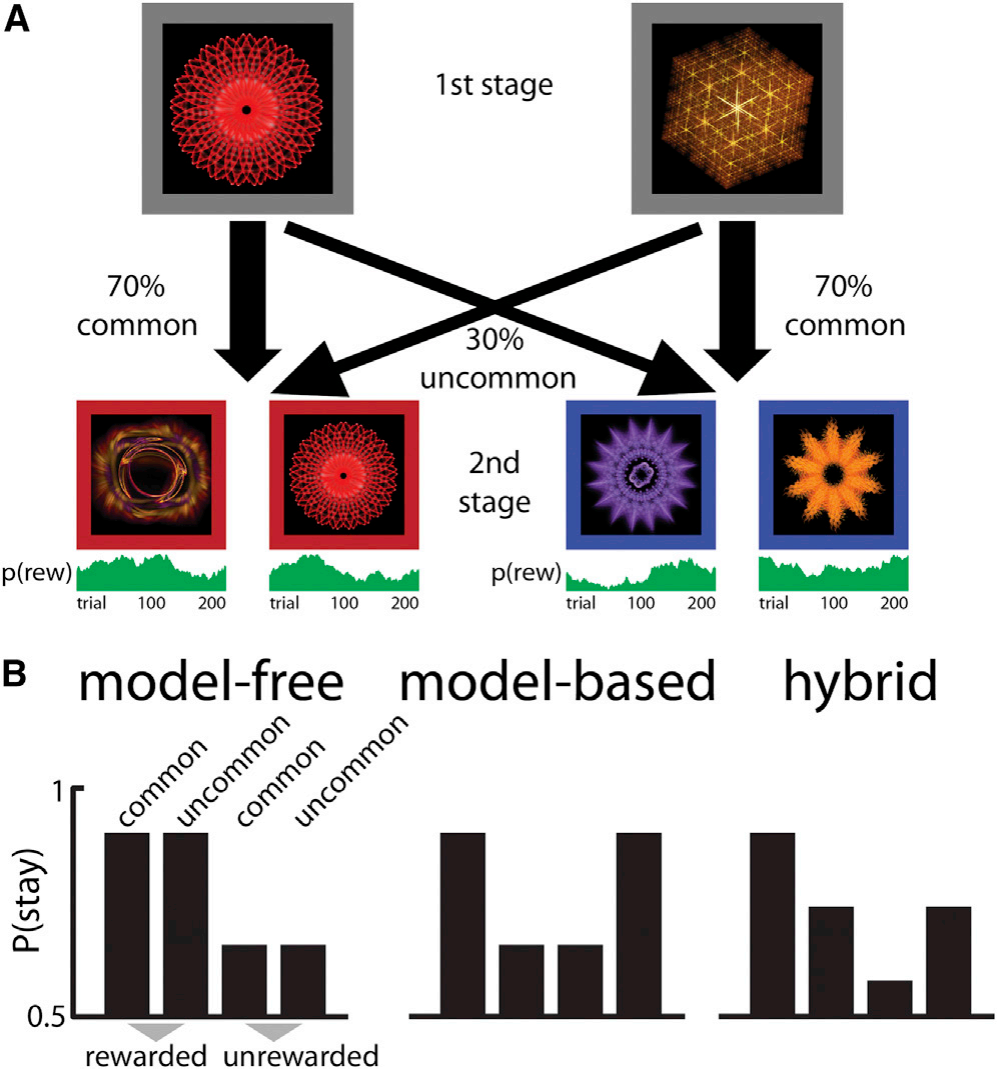
Task Design (A) On each trial, a choice between two stimuli led probabilistically to one of two further pairs of stimuli, which then demanded another choice followed by reward or no reward according to the p(reward) of the chosen second-stage stimulus that fluctuated over time. Importantly, participants could learn that each first-stage stimulus led more often (70%/30%) to one of the pairs; this task structure could then be exploited by a model-based, but not by a model-free, controller. (B) Model-based and model-free strategies for reinforcement learning predict differences in feedback processing particularly after uncommon transitions. If choices were exclusively model-free, then a reward would increase the likelihood of staying with the same stimulus on the next trial, regardless of the type of transition (left). Alternatively, if choices were driven by a model-based system, the impact of reward would interact with the transition type (middle). As shown previously, behavior in healthy participants resembles a hybrid of model-based and model-free control (right; Daw et al., 2011, Otto et al., 2013, Wunderlich et al., 2012b). We can thus quantify model-free control by estimating the main effect of reward, and model-based control by estimating the reward-by-transition interaction. Please see Figure S1 for a validation of the random walks.

We used a task that enables quantification of model-based and model-free control over choices (Daw et al., 2011). Participants were required to make two choices on every trial to arrive at a rewarded or a nonrewarded outcome (Figure 1A). Choices at the first stage of the task probabilistically determine which pair of options becomes available to the participant at the second stage. Crucially, for each first-stage action, one pair of second-stage options is more likely to occur (a “common transition”). Because a model-based controller is able to incorporate the probability of state-state transitions into its decision making, while the model-free controller is not, the predictions made by these controllers diverge after uncommon transitions, while being identical after common ones (Figure 1B). For example, a reward obtained after an uncommon transition prompts a model-free agent to (erroneously) choose the very same first-stage stimulus on the next trial, since action values are updated based solely on the reward that follows the action. In contrast, a model-based agent who can represent task structure would, upon receiving a reward after an uncommon transition, be more likely to switch to the previously unchosen first-stage stimulus, since this behavior is more likely to lead to the just-rewarded second-stage pair. Using these divergent predictions about first-stage choice behavior, we can infer the influence of the controllers in terms of the main effect of reward (model-free) and the interaction between reward and transition likelihood (model-based) on the probability of staying with the same first-stage stimulus (as in Daw et al., 2011). We refer to Figure S1 available online for a validation of this approach and Figure S2A for an analysis of second-stage choices.

**Figure 2:**
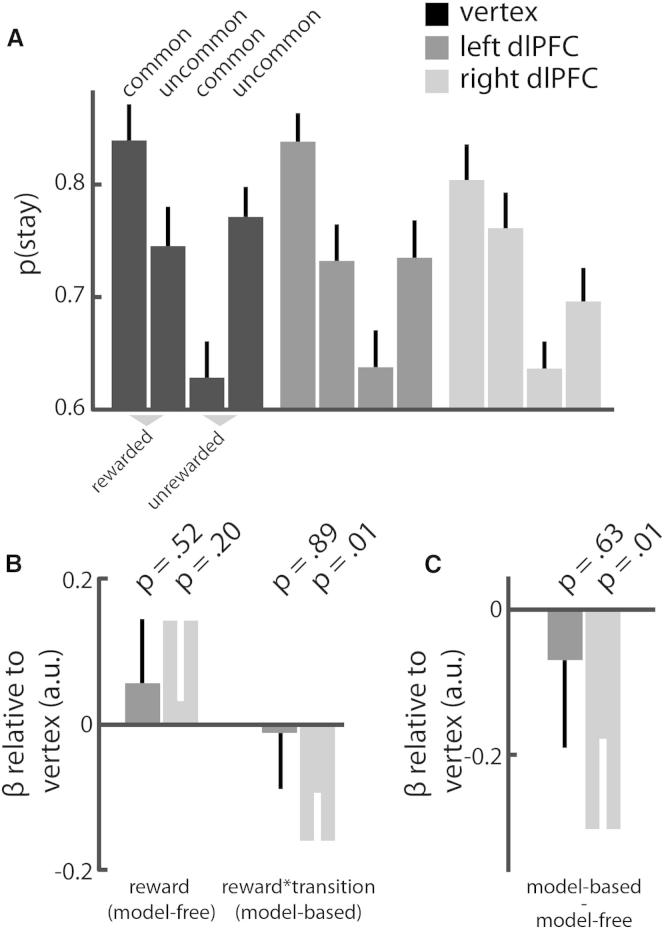
Results (A) The probability of repeating the same first-stage choice is shown as a function of reward and transition experienced on the previous trial. The pattern of choices qualitatively resembles influences of both model-based and model-free control for all three stimulation sites (cf. Figure 1B, right). (B) We quantified model-free and model-based control as the main effect of reward and the reward-by-transition interaction, respectively, in a hierarchical logistic regression on stay/switch behavior on each trial. Disruption of right dlPFC reduced model-based control compared to vertex. TBS did not significantly affect model-free control. (C) The relative balance between the controllers was calculated as β_model-based_ – β_model-free_. The balance significantly shifted toward model-free control after disruption of right, but not left, dlPFC compared to vertex. Error bars indicate SEM. Please see Figure S2 for additional stay-switch analyses.

Participants’ first-stage choices for all three TBS conditions qualitatively reflected a hybrid of model-based and model-free control (Figure 2A; cf. Figure 1B). We estimated the main effect of reward and the reward-by-transition interaction for each TBS site using hierarchical logistic regression, with all coefficients taken as random effects across participants (see Experimental Procedures for details). We observed positive coefficients for the reward and reward-by-transition regressors for all three TBS sites (all p < 0.006), confirming that behavior comprised a hybrid of model-free and model-based control (see Figure S2B). Levels of model-based and model-free control after left and right dlPFC TBS were then contrasted with vertex (Figure 2B). We observed that TBS to neither left (p = 0.52) nor right (p = 0.20) dlPFC significantly changed model-free control compared to vertex. By contrast, model-based control was disrupted following TBS to right (p = 0.01) but not left (p = 0.89) dlPFC compared to vertex. We observed no difference in model-based control between left and right dlPFC (p = 0.13).

We also computed a measure of the relative balance between these two systems as β_model-based_ − β_model-free_ (Figure 2C). This showed a significant shift toward model-free control caused by TBS to right (p = 0.01) but not left (p = 0.63) dlPFC compared to vertex. We observed no difference between left and right dlPFC (p = 0.11). Together, these results provide evidence that right dlPFC exerts a causal role in model-based control and show that the balance between model-based and model-free control can be manipulated through prefrontal disruption via TBS.

We repeated these analyses to examine order effects. In pairwise session comparisons, we found no effect of session on model-free or model-based control or on the balance between model-based and model-free control (all p > 0.14), except for a marginally significant increase in model-free control in session 3 compared to session 1 (p = 0.04).

Model-based control is thought to depend on a number of processes including prefrontal working memory (WM) capacity. Given that studies of WM report lateralized functionality (e.g., Mull and Seyal, 2001), we asked whether the magnitude of a TBS effect might be related to WM capacity. To examine such interindividual differences, we could not use the population parameter estimates obtained through the regression. Instead, we extracted the numerical magnitude of the main effect of reward, the reward-by-transition interaction, and the difference between the two from each subject’s average stay probability in each of the four reward/transition conditions in each stimulation condition.

**Figure 3:**
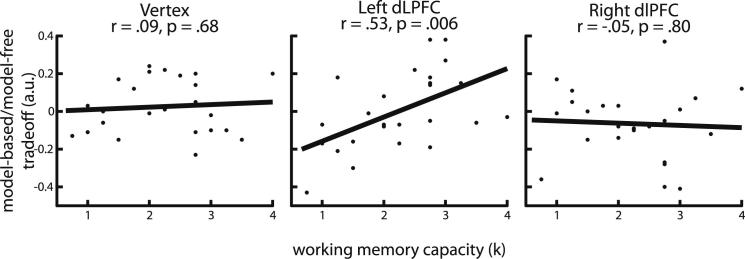
Working Memory Capacity Interacts with Stimulation in Left dlPFC Working memory (WM) capacity did not predict the balance between model-based and model-free control after disruption of vertex (left) or right dlPFC (right). In contrast, higher WM was associated with relatively stronger model-based control after disruption of left dlPFC (middle) with the correlation being significantly more positive than for right dlPFC (permutation test, p = 0.009) or vertex (p = 0.06).

We first asked whether model-free or model-based control independently correlated with WM in any of the three stimulation conditions. Only the magnitude of the reward-by-transition interaction, inferred as model-based control, correlated with WM after disruption to left dlPFC (r = 0.45, p = 0.02; all other p > 0.10). We then correlated the balance between the two systems in all stimulation conditions with WM. Strikingly, only behavior after disruption of left dlPFC was WM dependent (Figure 3; vertex, r = 0.09, p = 0.68; left dlPFC r = 0.53, p = 0.006; right dlPFC, r = −0.05, p = 0.80). Pairwise permutation tests revealed that the correlation was significantly more positive in left compared to right dlPFC (10^5^ permutations, p = 0.009), marginally more positive in left dlPFC compared to vertex (p = 0.06), and not significantly different between right dlPFC and vertex (p = 0.52). Taken together, these data show that the effect of left dlPFC disruption on the balance between model-based and model-free control depends on WM capacity, with high WM participants retaining more model-based control compared to those with low WM.

## Discussion

The balance between model-based and model-free control is often framed as a competition between a flexible, forward-looking system and a simpler retrospective stimulus-response-based system (Daw et al., 2005). Our results show that the balance between these two systems can be causally manipulated in the human brain by a disruption to prefrontal cortex. Our data suggest that TBS to right dlPFC impairs a key node in a network that underpins model-based control (cf. Gläscher et al., 2010, Killcross and Coutureau, 2003). We further show an involvement of left dlPFC in model-based control that is related to individual differences in working memory, suggesting differential roles for left and right dlPFC in the functional architecture underlying deliberative choice.

Animal lesion and human imaging work suggest that sectors of prefrontal cortex are involved in high-level cognition and decision making (Miller and Cohen, 2001). These studies have shown correlates of model-based control in ventromedial prefrontal cortex and dlPFC as well as outside the prefrontal cortex, e.g., dorsomedial striatum (Boorman et al., 2009, de Wit et al., 2009, Gallagher et al., 1999, Gläscher et al., 2010, Hikosaka, 2007, Killcross and Coutureau, 2003, Liljeholm and O’Doherty, 2012, Wunderlich et al., 2012a, Xue et al., 2012). In contrast, model-free control is most strongly associated with the dorsolateral striatum and infralimbic cortex (Balleine and O’Doherty, 2010, Wunderlich et al., 2012a, Yin et al., 2004). Furthermore, a strong dependence of model-based control on prefrontal systems is hinted by a finding that its dominance can be abolished during dual-task performance (Otto et al., 2013). However, up to now the key human evidence for dlPFC involvement in model-based control has been based on correlational evidence using functional imaging (fMRI). Here we show that model-based control is impaired by a transient disruption of the right dlPFC, providing causal evidence for its involvement in complex, flexible, decision making. We note that this effect was significant only when compared to the vertex, our control site, but not when compared to left dlPFC. We speculate that this might be due to individual variation in the role of the left dlPFC in model-based control or in the strategies employed by our participants to solve the task.

An influential hypothesis about the balance between model-based and model-free control states that their individual influence over behavior is governed by their respective uncertainties (Daw et al., 2005). Within this framework, our results can be interpreted as emerging out of a disruption to a key component process of model-based control (e.g., the utilization of associative models; Gläscher et al., 2010). This would lessen the certainties of model-based predictions leading to an attenuated dominance over behavior—similar to that observed when subjects are distracted by a dual task (Otto et al., 2013). However, whereas disruption of right dlPFC led to an unambiguous impairment of model-based control, the effect of TBS on the left dlPFC was dependent on baseline WM capacity. Specifically, higher WM capacity conferred a degree of protection against a shift toward model-free control upon disruption of left dlPFC, whereas participants with low WM capacity appear to require an uncompromised left dlPFC for the exercise of model-based control. We acknowledge uncertainty as to what precise factors might explain this finding.

We note that TBS to left, but not right, dlPFC has been reported to decrease dopamine levels across the basal ganglia (Ko et al., 2008). This effect might interact with baseline dopamine levels that are known to covary with WM capacity (Cools et al., 2008), such that high WM participants are more resilient against TBS-induced decreases in dopamine than low WM participants. We previously showed that dopamine levels modulate the balance between model-based and model-free control (de Wit et al., 2011, de Wit et al., 2012, Wunderlich et al., 2012b), and a TBS-induced depletion in low WM (i.e., low dopamine) individuals might have a more pronounced effect than a similar depletion in high WM (i.e., high dopamine) individuals. However, given that we did not directly measure dopamine levels, future work could usefully explore potential interactions between WM and model-based control to fully understand the effect reported here.

Our findings speak to the literature on goal-directed and habitual behaviors (Balleine and O’Doherty, 2010). Although model-based/model-free and goal-directed/habitual control are not synonymous, the former provides a computational framework that can encompass key features of goal-directed and habitual control (for a review, see Dayan and Niv, 2008). We would predict that a disruption of right dlPFC would also impair goal-directed behavior in devaluation and contingency degradation tests in humans, as has been shown in rats (Balleine and O’Doherty, 2010).

In summary, we provide causal evidence for a role of the right dlPFC in flexible, model-based decision making. Our findings invite the question as to whether naturally occurring variation in dlPFC function and connectivity is a marker for predisposition toward model-free as opposed to model-based control and whether an enhancement of dlPFC function (e.g., through other stimulation protocols) might improve rather than impair model-based control.

## Experimental Procedures

### Participants

Twenty-five adults participated in the experiment (15 females; age range 18–35 years; mean = 24.2, SD = 4.0 years). All participants had normal or corrected-to-normal vision and were without a history of psychiatric or neurological disorder. All participants provided written informed consent prior to start of the experiment, which was approved by the Research Ethics Committee at University College London (UK). No participants were excluded over the course of the experiment.

### General Design

Participants were tested on 3 days between 3 and 16 days (mean = 5.9, SD = 2.6) apart. In each session, participants practiced 50 trials of the task before receiving offline theta burst transcranial magnetic stimulation (TBS; Huang et al., 2005) to the right dorsolateral prefrontal cortex (dlPFC), left dlPFC, or vertex. Participants then performed 201 trials on the task.

### Task

The task design was based on Daw et al. (2011) and identical to Wunderlich et al. (2012b) except for faster trial timings to fit the task within a constraint of 20 min, i.e., the estimated time during which TBS modulates local neuronal excitability (Huang et al., 2005). The task was programmed in Cogent 2000 & Graphics (John Romaya, Wellcome Trust Centre for Neuroimaging and Institute of Cognitive Neuroscience development team, UCL) in MATLAB (MathWorks).

Each trial consisted of two choice stages. Each choice stage contained a two-alternative forced choice, with choice options represented by a fractal in a colored box on a black background (Figure 1A). On each choice, participants had to respond within 2 s using the left/right cursor keys or the trial was aborted and reward omitted. Missed trials (mean = 0.1%, range = 0%–1.5%) were omitted from analysis.

Choice at the first stage always involved the same two stimuli. After participants made their response, the rejected stimulus disappeared from the screen and the chosen stimulus moved to the top of the screen. After 0.5 s, one of two second-stage stimulus pairs appeared, with the transition from first to second stage following fixed transition probabilities. Each first-stage option was more strongly (with a 70% transition probability) associated with one of the two second-stage pairs, a crucial factor in allowing us to distinguish model-free from model-based behavior (see below). In both stages, the two choice options were randomly assigned to the left and right side of the screen, forcing the participants to use a stimulus- rather than action-based learning strategy. After the second choice, the chosen option remained on the screen, together with a reward symbol (a pound coin) or a “no reward” symbol (a red cross). Each of the four stimuli in stage two had a reward probability between 0.2 and 0.8. These reward probabilities drifted slowly and independently for each of the four second-stage options through a diffusion process with Gaussian noise (mean 0, SD 0.025) on each trial. Three random walks were generated beforehand and randomly assigned to sessions. We chose to preselect random walks as otherwise they might, by chance, turn out to have relatively static optimal strategies (e.g., when a single second-stage stimulus remains at or close to p(reward) = 0.8). Such static optimal strategies can lead to the emergence of a reward-by-transition interaction even in a purely model-free agent due to the nature of the 1-back regression analysis (also see Figure S1 for a validation of our random walks).

Prior to the experiment, participants were explicitly instructed that for each stimulus in the first stage, one of the two transition probabilities was higher than the other and that these transition probabilities remained constant throughout the experiment. Participants were also told that reward probabilities on the second stage would change slowly, randomly, and independently over time. On all 3 days, participants practiced 50 trials with different stimuli before starting the task. The main task consisted of 201 trials with 20 s breaks after trial 67 and 134. The participant’s payment was determined as a flat rate plus their overall accumulated reward from both sessions. Reward per session ranged from 3.75–12.75 in £s (mean = 8.4, SD = 2.4; no difference between sessions [F(2,48) = 1.51, p = 0.23] or TBS sites [F(2,48) = 1.23, p = 0.30] in three-way ANOVA).

### Baseline Working Memory Capacity

In the first session, before any TBS or practice on the main task, participants performed a 7 min task to establish visuospatial working memory capacity. In short, participants had to remember the location of five simultaneously presented dots in a circular array of 16 positions. After a delay, the participant was asked whether, for one of the 16 locations, a red dot was presented. From these data, we calculated a K value, reflecting the amount of information that the participant can store in working memory. For details of the task and analysis, see McNab and Klingberg (2008).

### Theta Burst Stimulation

Participants received TBS over the right dlPFC, left dlPFC, and vertex on three separate occasions, with site order counterbalanced across 24 participants, and the 25^th^ participant received a randomly selected session order. We identified stimulation sites as follows: the MNI coordinates for the right dlPFC (x = 37, y = 36, z = 34) were taken from a previous study that used a combination of individual anatomy and fMRI results to pinpoint the dlPFC (Feredoes et al., 2011). For the left dlPFC (x = −37, y = 36, z = 34), we took the negative of the right dlPFC x-coordinate. These MNI coordinates were transformed to coordinates in native space by taking the inverse normalization parameters from unified segmentation of a previously acquired T1w structural image as implemented in SPM8 (Wellcome Trust Centre for Neuroimaging, UCL, UK). We visually confirmed that the coordinates in native space corresponded to middle frontal gyrus (as in Feredoes et al., 2011). These coordinates were then entered as targets into Visor2 (ANT B.V.), which uses a 3D camera to guide the stimulation coil (Magstim) to the target coordinate. The vertex was set to the Cz of the 10-20 system. To mimic the stimulation experience for the participant, we entered the vertex coordinates into Visor2 and used 3D navigation to target the stimulation coil.

We administered stimulation in 5 Hz bursts of three pulses set 20 ms apart, for 40 s, amounting to a total of 600 pulses. Stimulation intensity was set for each individual participant as 90% of active motor threshold (AMT). AMT was defined as the lowest stimulation intensity, expressed as a percentage of max output of the Magstim equipment that reliably (3/5 times) yielded a visible muscle twitch in the hand when stimulating the hand area of the contralateral motor cortex with a single pulse. During this procedure, participants held (lightly) an item in the hand contralateral to the stimulation site. For technical and safety reasons, the maximum stimulation intensity was set to 51% of maximum output; as such, any participant with an AMT > 56% received TBS at 51% of maximum output. Note that such reduced stimulation will make it less likely to find significant effects of TBS. The average stimulation intensity was 49% (range: 40%–51%) of maximum output.

### Analysis

We analyzed stay-switch behavior on the first choice of each trial to dissociate model-based and model-free control. A model-free reinforcement learning strategy predicts a main effect of reward on stay probability. This is because model-free choice works without considering structure in the environment; hence, rewarded choices are more likely to be repeated, regardless of whether that reward followed a common or rare transition (Figure 1B, left). A reward after an uncommon transition would therefore adversely increase the value of the chosen first stage cue without updating the value of the unchosen cue. In contrast, under a model-based strategy, we expect an interaction between transition and reward on the previous trial, because a rare transition inverts the effect of a subsequent outcome (Figure 1B, middle). Under model-based control, receiving a reward after an uncommon transition increases the propensity to switch. This is because the rewarded second-stage stimulus can be more reliably accessed by choosing the rejected first-stage cue than by choosing the same cue again. To summarize, this analysis quantifies model-free behavior as the strength of the main effect of reward and model-based behavior as the strength of the reward by transition interaction, even when actual behavior is a hybrid of model-free and model-based control (Figure 1B, right).

We used hierarchical logistic regression implemented in lme4 (Bates et al., 2012) in the R software package (R Development Core Team, 2011). We estimated coefficients for the regressors shown in Table 1, taking all coefficients as random effects over participants. This method accounts for both within- and between-subject variance, providing unbiased estimates of the population coefficient for each regressor. We then performed contrasts over the population coefficients to test for differences between conditions in model-free and model-based control. All p values reported in the manuscript that pertain to the logistic regression are based on the chi-square distribution and were estimated using the “esticon” procedure in the “doBy” package (Højsgaard, 2006).

**Table 1:** Regressors for Hierarchical Logistic Regression

Intercept
Left dlPFC
Right dlPFC
Left dlPFC × reward
Right dlPFC × reward
Vertex × reward
Left dlPFC × transition
Right dlPFC × transition
Vertex × transition
Left dlPFC × reward × transition
Right dlPFC × reward × transition
Vertex × reward × transition

Regressors for hierarchical logistic regression on stay (coded as 1) or switch (coded as 0) for each first-stage choice. The main effect of vertex is subsumed in the intercept.
