# Supplementary material for: Disruption of Dorsolateral Prefrontal Cortex Decreases Model-Based in Favor of Model-free Control in Humans
Source: Neuron. 2013 Nov 20;80(4):914–9. doi: 10.1016/j.neuron.2013.08.009 (PMC3893454; doi:10.1016/j.neuron.2013.08.009)
Supplement: Document S1. Figures S1 and S2 [file mmc1.pdf]

Neuron, Volume *80*

Supplemental Information

Disruption of Dorsolateral Prefrontal Cortex Decreases Model-Based in Favor of Model-free Control in Humans

Peter Smittenaar, Thomas H.B. FitzGerald, Vincenzo Romei, Nicholas D. Wright, and Raymond J. Dolan

Supplemental information

Supplemental figure 1 (related to Figure 1): Validation of random walks

A purely model-free agent can, under restricted circumstances, generate data that contains a reward-by-transition interaction in the 1-back analysis employed here. Such a confound arises when the second-stage reward probabilities are relatively static, because the participant might settle on the best first-stage stimulus rather than switch between the two first-stage stimuli on a regular basis. This would then lead to a situation where most common transitions are rewarded, and most uncommon transitions are unrewarded, when choosing the best stimulus. In both cases, the participant likely stays with the same first-stage stimulus on the first trial, even if the participant is completely model-free, thus leading to a reward-by-transition interaction that is inferred as model-based control. This confound is quickly alleviated when the second-stage reward probabilities become less static, or indeed follow random walks. In our experiment we used 3 random walks randomly assigned to sessions. To confirm that these walks were not confounded we generated data from a model-free agent playing on our random walks, and independently of these simulations by repeating the hierarchical regression with an added regressor that captures this confound.

First, we generated data using a reinforcement learning model identical to that used by Otto et al. (2013), which has 5 parameters: a model-free learning rate shared between the first and second stage (α), inverse temperature for the softmax choice rule (β), eligibility trace which carries the second-stage prediction error over to the first-stage stimuli (λ), a weighting parameter between model-based and model-free control (ω) and a perseverance parameter which accounts for a propensity to stay regardless of previous events (π). For details of the model please see Otto et al. (2013). We selected representative values for all but α: $\beta = 4$, $\lambda = 0.6$, $\omega = 0$ (i.e. purely model-free), $\pi = 0.1$ (Daw et al., 2011). As the potential confound can depend on α, we generated data for α between 0.001 and 0.600 in steps of 0.001, simulating 3000 datasets of 201 trials for each configuration of parameters. We then calculated p(stay) for each of the four reward/transition conditions and calculated the magnitude of the main effect of reward and reward-by-transition interaction from these p(stay) values. Note this is a different approach from the hierarchical analysis, but along identical lines of reasoning. Crucially, we predicted that a model-free agent should not show any reward-by-transition interaction in any of the walks, as that would indicate a potential confound in the walks. As expected, these walks did not show such a confound (Figure S1), such that the level of model-based control was close to zero for all learning rates. Interestingly, the level of inferred model-free control scaled close to linear with learning rate. In conclusion, in our random walks our estimation of model-based control is not confounded by model-free influences on behavior.

Second, we accounted for the potential confound in the hierarchical regression by regressing out variance in stay behavior accounted for by simply choosing the stimulus that leads, for common transitions, to the highest-value second-stage stimulus. We added a regressor that was +1 if the chosen first-stage stimulus was commonly associated with the current best second-stage stimulus, and -1 if the other stimulus was chosen.

Although this regressor was significantly positive (estimate ± SE: 0.12 ±0.03, $p = .0004$), its inclusion only minimally affected the estimation of the other regressors. All estimates and contrasts reported

as significant remained so, and all reported non-significant remained so too. Most notable, this same model still showed that model-based control was disrupted after TBS to right (p = .01) but not left (p = .63) dlPFC compared to vertex (left versus right, p = .09). Behavior shifted significantly away from model-based towards model-free control after TBS to right (p = .009) but not left (p = .63) dlPFC compared to vertex (left versus right, p = .09).

These two analyses confirm that all three random walks were not susceptible to a potential confound whereby purely model-free control leads to a pattern of behavior that is interpreted as model-based.

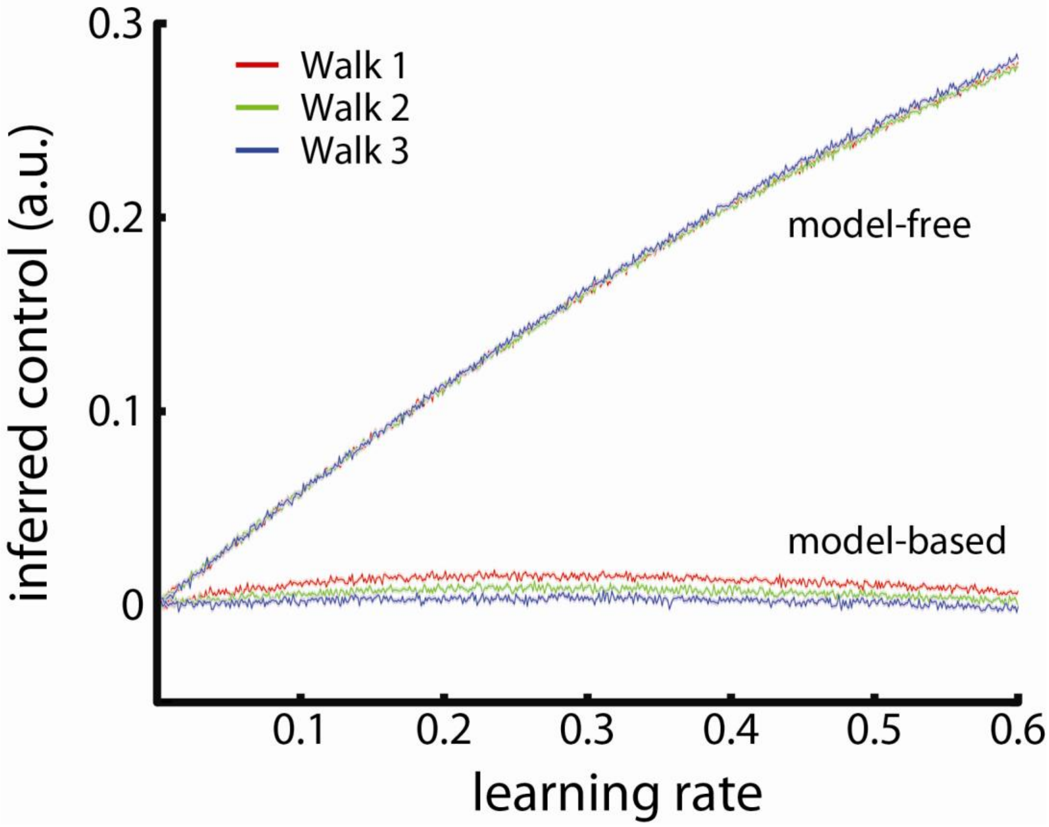

Figure S1: inferred level of model-based and model-free control in a purely model-free agent as a function of learning rate of this agent. We simulated 3000 agents playing this task for every learning rate to verify that our analysis method would not infer model-based control even though the underlying generative model was purely model-free. For all three walks used in our experiment, the analysis correctly estimated model-based control to be around zero irrespective of the learning rate.

Supplemental figure 2 (related to Figure 2): Additional stay-switch analyses

Second-stage choices

Whereas first-stage choices allow us to dissociate model-based from model-free control, both types of control make equivalent predictions for second-stage choices as there is no task structure to exploit. It has, however, been shown that TBS to left, but not right, dlPFC modulates probabilistic instrumental reward learning (Ott et al., 2011). We therefore sought to explore the effects of TBS on 1-step reward learning here as well (Figure S2A). We examined second-stage choices using

hierarchical logistic regression similar to our analysis of first-stage choices: stay-switch behavior was regressed against reward received on the most recent trial involving that second-stage pair. Transition was not included as a factor because second-stage choices are assumed to be independent of the transition type that led to the state. We observed that TBS to left dlPFC affected second-stage choices by making them more perseverative (p = .02) and more sensitive to reward (p = .006) compared to vertex (see figure S2A). No such effect was found for right dlPFC (p = .11 and p = .10, respectively). There was no difference between left and right dlPFC (perseveration: p = .35, effect of reward: p = .20). An increase in perseveration might be caused by a reduction in striatal dopamine after TBS to left dlPFC (Ko et al., 2008), which is known to affect behavioral flexibility and perseverance (Cools et al., 2006). It is, however, unclear why such a reduction in striatal dopamine would be associated with *improved* reward learning. We note this finding replicates a previous study that observed improved reward learning after TBS to left, but not right, dlPFC (Ott et al., 2011). Arguing against a role for dopamine in this increase in reward sensitivity is a null effect of levodopa administration on second-stage choices shown previously (Wunderlich et al., 2012).

Absolute regression coefficients for first-stage choices

Here we provide the estimates for the 6 population coefficients of interest in the hierarchical logistic regression (i.e. the absolute values from which the contrasts in Figure 2B and 2C are derived; Figure S2B). All coefficients were significantly larger than zero, indicating that behavior in all three conditions was a hybrid of model-free and model-based control (main effect of reward: vertex $p = 8.9 \times 10^{-4}$; left dlPFC $p = 1.4 \times 10^{-5}$, right dlPFC $p = 1.5 \times 10^{-6}$; reward-by-transition interaction: vertex $p = 2.9 \times 10^{-8}$, left dlPFC $p = 5.6 \times 10^{-4}$, right dlPFC p = .006).

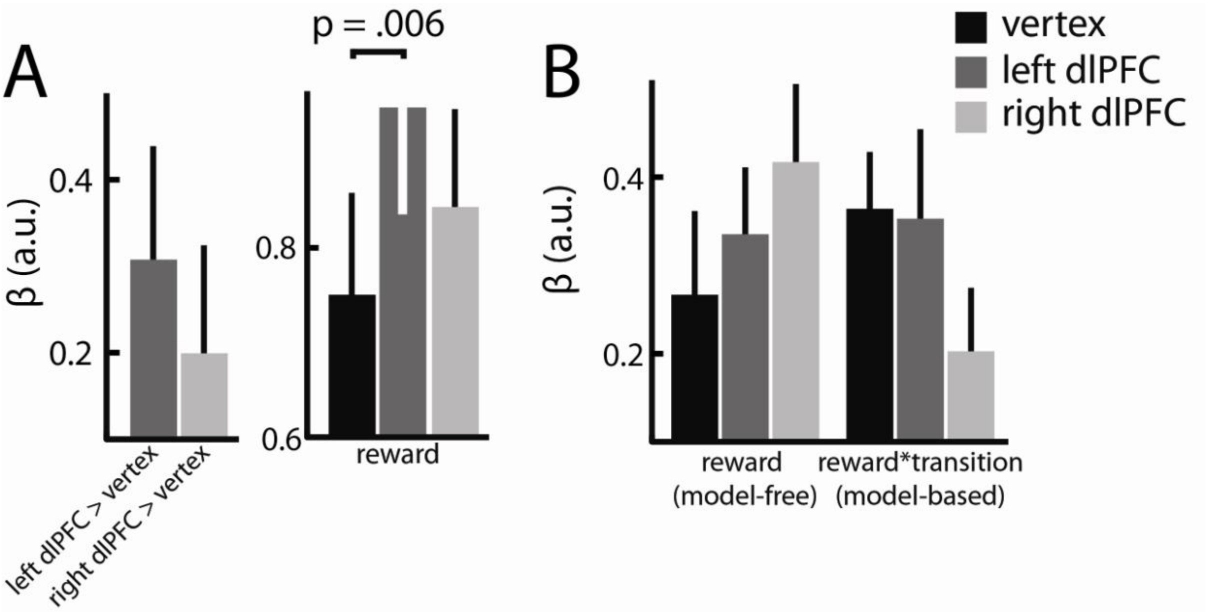

Figure S2: complementary stay-switch analyses, supplemental to Figure 2. (A) Analysis of second-stage choices. The main effect of each stimulation site (left) captures the propensity to stay with the same stimulus irrespective of reward, relative to the vertex condition. Participants become more perseverative after left dlPFC TBS compared to vertex (p = .02) on second-stage choices. Note that the main effect of vertex is subsumed in the intercept of the regression, such that a coefficient

significantly different from zero indicates a significant deviation from vertex. The main effect of reward in each stimulation condition(right) indicated participants tended to stay with a rewarded stimulus more than with an unrewarded stimulus (all p < .001), but this propensity was stronger after left dlPFC TBS compared to vertex (p = .006). (B) Coefficients for first-stage choices. Both the main effect of reward and the reward-by-transition interaction were positive in all three TBS conditions, indicating the presence of both model-free and model-based influences on behavior.

References

Cools, R., Ivry, R.B., and D'Esposito, M. (2006). The human striatum is necessary for responding to changes in stimulus relevance. J Cogn Neurosci 18, 1973-1983.

Daw, N.D., Gershman, S.J., Seymour, B., Dayan, P., and Dolan, R.J. (2011). Model-based influences on humans' choices and striatal prediction errors. Neuron 69, 1204-1215.

Ko, J.H., Monchi, O., Ptito, A., Bloomfield, P., Houle, S., and Strafella, A.P. (2008). Theta burst stimulation-induced inhibition of dorsolateral prefrontal cortex reveals hemispheric asymmetry in striatal dopamine release during a set-shifting task: a TMS-[(11)C]raclopride PET study. Eur J Neurosci 28, 2147-2155.

Ott, D.V.M., Ullsperger, M., Jocham, G., Neumann, J., and Klein, T.A. (2011). Continuous theta-burst stimulation (cTBS) over the lateral prefrontal cortex alters reinforcement learning bias. NeuroImage 57, 617-623.

Otto, A.R., Gershman, S.J., Markman, A.B., and Daw, N.D. (2013). The curse of planning: dissecting multiple reinforcement-learning systems by taxing the central executive. Psychol Sci 24, 751-761.

Wunderlich, K., Smittenaar, P., and Dolan, R.J. (2012). Dopamine enhances model-based over model-free choice behavior. Neuron 75, 418-424.
